# Irisin modulates the transcriptomic profile of porcine anterior pituitary cells isolated from gilts on days 15–16 of pregnancy

**DOI:** 10.1038/s41598-026-51519-6

**Published:** 2026-05-12

**Authors:** Barbara Zarzecka, Kamil Dobrzyn, Marta Kiezun, Grzegorz Kopij, Katarzyna Kisielewska, Agnieszka Rak, Tadeusz Kaminski, Nina Smolinska

**Affiliations:** 1 Department of Animal Anatomy and Physiology, Faculty of Biology and Biotechnology, University of Warmia and Mazury in Olsztyn, Oczapowskiego St. 1A, 10-719 Olsztyn, Poland; 2 Doctoral School of the University of Warmia and Mazury in Olsztyn, Oczapowskiego St. 5, 10-719 Olsztyn, Poland; 3 Department of Molecular Biology of Reproduction, InLife Institute of Animal Reproduction and Food Research, Polish Academy of Sciences, Trylinskiego St. 18, 10-683 Olsztyn, Poland; 4 Department of Anatomy and Histology, School of Medicine, University of Warmia and Mazury in Olsztyn, Warszawska 30, 10-082 Olsztyn, Poland; 5 Laboratory of Physiology and Toxicology of Reproduction, Institute of Zoology and Biomedical Research, Faculty of Biology, Jagiellonian University, Gronostajowa St. 9, 30-387 Kraków, Poland

**Keywords:** Irisin, RNA-Seq, Anterior pituitary, Differentially expressed genes, Pregnancy, Pig, Developmental biology, Endocrinology, Genetics, Molecular biology, Physiology

## Abstract

Irisin, an adipomyokine primarily known for its role in metabolic regulation, has recently emerged as a potential regulator of reproductive function. While its ovarian actions have been partially characterised, its influence on the anterior pituitary remains largely unexplored. This study aimed to investigate the in vitro effects of irisin on the transcriptome of porcine anterior pituitary cells (APc) isolated from gilts on days 15–16 of pregnancy. Primary APc were isolated from pigs on days 15–16 of pregnancy and treated with 300 ng/mL of irisin. RNA sequencing (NovaSeq 6000, Illumina) was performed to identify differentially expressed genes (DEGs), long non-coding RNAs (lncRNAs), and alternative splicing (AS) events. Functional annotation was performed using Gene Ontology (GO) and Kyoto Encyclopedia of Genes and Genomes (KEGG) analyses. Selected transcripts were validated by polymerase chain reaction (PCR) and quantitative PCR (qPCR). Irisin treatment altered the expression of 500 genes, 747 lncRNAs, and 245 transcripts undergoing AS events. Differentially expressed genes were enriched in pathways related to hormonal signalling, angiogenesis, calcium signalling, and cell remodelling. Notably, genes encoding key regulators of these processes, such as growth hormone 1, insulin-like growth factor type 1, somatostatin, prolactin receptor, and progesterone receptor, were modulated. Pathway enrichment analysis indicated enrichment of genes assigned to Janus kinase/signal transducer and activator of transcription, phosphoinositide 3-kinase/protein kinase B, and mitogen-activated protein kinase signalling cascades. This study provides the first comprehensive transcriptomic profiles of irisin action on porcine APc during early pregnancy. Irisin can modulate endocrine, structural, and molecular functions through both transcriptional and post-transcriptional mechanisms, positioning it as a novel modulator of pituitary adaptation in early gestation.

**Supplementary Information:**

The online version contains supplementary material available at 10.1038/s41598-026-51519-6.

## Introduction

Adipokines are biologically active molecules secreted by adipose tissue that play a key role in regulating reproductive processes and pituitary function^1^. Similarly, myokines, hormones primarily produced by skeletal muscle, also exert regulatory effects on both metabolic and reproductive functions^2,3^. Both adipokines and myokines function as endocrine mediators of energy homeostasis; yet, accumulating evidence suggests their broader involvement in reproductive regulation^4–8^. These molecules can act directly or indirectly on the components of the hypothalamic-pituitary-gonadal (HPG) axis, a crucial system controlling reproductive physiology^1^. Irisin, a relatively recently discovered hormone by Boström et al.^9^, is classified as an adipomyokine, produced by both adipose and muscle tissues. It is a 112–amino acid peptide with a molecular mass of approximately 12.6 kDa, cleaved from the fibronectin type III domain-containing protein 5 (FNDC5) precursor^9^. Irisin has been linked to the maintenance of metabolic homeostasis and is involved in thermogenesis, the browning of white adipose tissue, improved insulin sensitivity and enhanced glucose metabolism^10–16^. A growing body of literature points to its potential role in female fertility and overall reproductive health^8^.

The HPG axis is the central regulatory system for reproductive endocrinology. Within this axis, the anterior pituitary gland plays a pivotal role by secreting hormones that govern reproduction, growth, metabolism, and stress responses^17,18^. During pregnancy, the pituitary undergoes dynamic morphological and functional changes, including gland enlargement due to lactotroph hyperplasia and hypertrophy^19^, accompanied by progressive increases in prolactin (PRL) secretion^20,21^. Considering the pituitary’s central role in reproductive regulation and its significant remodelling during gestation, identifying factors, such as irisin, that may modulate pituitary activity is of particular importance. Emerging evidence suggests that irisin may influence the HPG axis by modulating gonadotrophins and reproductive hormone production, which are essential for female fertility. It is particularly implicated in metabolic adaptations during pregnancy^3,22^. Studies have shown higher circulating levels of irisin in females compared to males^23^. Moreover, during normal pregnancy, circulating irisin concentrations are elevated compared with the non-pregnant state and positively correlate with insulin resistance in early gestation^3^. Conversely, lower maternal irisin levels in the first trimester have been associated with a higher risk of developing gestational diabetes mellitus^24,25^.

The presence of irisin in the pituitary gland has been confirmed across several species, including tilapia^26^, mole rats^27^, cattle^8^, humans^28^, and pigs^29^. Our recent studies revealed that its levels vary depending on the hormonal status of the pig^29^. In the porcine pituitary cell cultures, supplementation with gonadotrophin-releasing hormone (GnRH), luteinising hormone (LH), or follicle-stimulating hormone (FSH) suppresses irisin secretion^29^. In mouse hypothalamic GT1-7 cells, irisin stimulates GnRH release in vitro^23^. In the pituitary of rats, bovine, and in the mouse mPit12 cell line, irisin also increases FSH and LH expression^30–32^. Moreover, global deletion of the *FNDC5* gene in female mice leads to disturbed oestrous cycles, ovarian dysfunction, morphological abnormalities, and reduced plasma concentrations of key reproductive hormones, including oestradiol, FSH, and LH^2^. Irisin deficiency has additionally been associated with lower body weight and increased mortality in mice^2^. Collectively, these findings support the hypothesis that irisin is an important modulator of female reproductive physiology at the pituitary level.

We hypothesised that irisin may influence the expression of a broader spectrum of genes whose products may be involved, directly or indirectly, in the regulation of reproductive processes. Accordingly, we focused on days 15–16 of gestation (the beginning of implantation) when conceptus-derived oestrogens provide the maternal recognition signal and elevated progesterone (P4) and oestradiol strongly constrain GnRH and LH pulsatility, rendering the anterior pituitary particularly sensitive to the hormonal milieu. In the present study, we investigated the global transcriptomic response to irisin in porcine anterior pituitary cells (APc), isolated from gilts on days 15–16 of gestation, focusing on differentially expressed genes (DEGs), long non-coding RNAs (lncRNAs) expression, and alternative splicing (AS) events.

## Methods

### Experimental animals and tissue collection

The study was conducted following the ethical standards outlined in the Polish Act on the Protection of Animals Used for Scientific or Educational Purposes (January 15th 2015; Journal of Laws 2015 No. item 266) and Directive 2010/63/EU of the European Parliament and Council (September 22^nd^ 2010) on the protection of animals used for scientific purposes. Animals were slaughtered in a commercial abattoir according to standard meat production procedures (EU Regulation No. 1099/2009). All tissues used in this study were collected post mortem from a commercial slaughterhouse during routine meat production. No experimental procedures were performed on live animals. Therefore, approval from an ethics committee was not required. Crossbred mature gilts (7–8 months old, 140–150 kg) on days 2–3, 10–12, 14–16, and 17–19 of the oestrous cycle and 15–16 of gestation (*n* = 5 per group) were obtained from a private breeding farm. Oestrous behaviour was monitored daily in the presence of a boar, and the onset of the second oestrus was designated as day 0. Natural mating occurred on days 1–2 of the oestrous cycle. Immediately after slaughter, blood, pituitary glands, and uteri were collected and placed in ice-cold Dulbecco’s phosphate-buffered saline supplemented with 100 IU/mL penicillin and 100 µg/mL streptomycin. Tissues were transported on ice to the laboratory for further analysis. The stage of the oestrous cycle was determined based on ovarian morphology, according to the criteria described by Akins and Morrissette^33^. Pregnancy status was confirmed by flushing the uterus and assessing the presence and morphology of conceptuses, according to the criteria described by Anderson^34^.

### Determination of irisin concentration in pig plasma

Blood samples were collected from pregnant gilts at slaughter and centrifuged to obtain plasma, which was stored at − 20 °C until analysis. Plasma irisin concentrations were determined using a commercially available enzyme-linked immunosorbent assay (ELISA) Kit (catalogue no. EK-067-29; Phoenix Pharmaceuticals, Inc., USA). Plasma samples were obtained from gilts on days 10–11, 12–13, 15–16, and 27–28 of pregnancy (*n* = 5 per group). Each sample was analysed individually, and irisin concentrations were expressed as ng/mL. The standard curve ranged from 0.1 to 1000 ng/mL, with a test sensitivity of 1.29 ng/mL. Intra- and inter-assay coefficients of variation were < 10% and < 15%, respectively. Absorbances were measured at 450 nm by using Infinite M200 PRO reader with Tecan i-control software (Tecan, Männedorf, Switzerland). The results are presented as mean ± SD., and raw individual values were additionally provided in the Supplementary Table. Mean plasma irisin concentrations were 222.31 ± 53.98 ng/mL on days 10–11, 241.77 ± 44.09 ng/mL on days 12–13, 227.96 ± 44.04 ng/mL on days 15–16, and 206.54 ± 34.72 ng/mL on days 27–28 of pregnancy.

### In vitro culture of APc

The anterior and posterior pituitary lobes were separated manually before digestion based on clear macroscopic boundaries visible in freshly collected porcine pituitaries. This anatomical approach is additionally supported by our previous studies in the porcine pituitary, in which immunofluorescence analyses demonstrated colocalisation of pituitary hormones with irisin^29^ and visfatin^35^ in anterior pituitary tissue collected using the same lobe-separation procedure, supporting the anatomical identification of the material used for APc isolation. Isolation of APc followed the protocol described by Szymańska et al.^35^. Each gland (*n* = 5) was rinsed in Dulbecco’s medium (Merck, USA) containing 0.1% bovine serum albumin and 1% Antibiotic-Antimycotic Solution (containing penicillin, streptomycin, amphotericin B) (Merck, USA). Tissues were minced with a scalpel and digested with 0.2% collagenase (Merck, USA) at 37 °C for 30 min. The resulting single-cell suspension was centrifuged three times at 1000 × g for 10 min. Remaining tissue fragments underwent a second enzymatic digestion using 0.2% collagenase and 0.25% pancreatin (Merck, USA) until fully dissociated. The suspension was filtered through a 70 μm nylon mesh (Greiner, Austria) to remove debris. Cell number was determined using a hemocytometer, and viability was assessed by Trypan Blue (Merck, USA) exclusion. All isolations yielded ≥ 90% viable cells. Cells were seeded into 6-well culture plates at a density of 1.5 × 10^6^ cells/2 mL/well in McCoy’s-5 A medium (Merck, USA) supplemented with 10% horse serum (Merck, USA) and 2.5% foetal bovine serum (Merck, USA). Pre-incubation was carried out at 37 °C in a humidified atmosphere of 5% CO_2_ and 95% air for 72 h. After 48 h, 1 mL of serum-free culture medium was added, followed by an additional 24 h incubation. Subsequently, medium was replaced with fresh serum-free McCoy’s-5 A medium containing 300 ng/mL irisin (recombinant human FNDC5, product No. APN576Hu01, Cloud-Clone Corp. USA). Based on the plasma irisin concentration measured on days 15–16 of pregnancy (227.96 ± 44.04 ng/mL), 300 ng/mL was selected as an upper-range physiologically relevant concentration for the in vitro experiments. Control wells received medium without irisin. Cells were cultured for a further 24 h before total RNA extraction.

### Dose-response viability assay

To preliminarily evaluate whether irisin affects the viability of porcine anterior pituitary cells (APc), a pilot dose-response experiment was performed using primary APc isolated from gilts on days 2–3, 10–12, 14–16, and 17–19 of the oestrous cycle. Cells were isolated and diluted into 96-well plates (100,000 cells/well) and cultured as described above. After preincubation, the culture medium was replaced with fresh serum-free medium containing irisin at concentrations of 100, 200, or 300 ng/mL; control wells received medium without irisin. Cells were then incubated for 24 h.

Cell viability was assessed using the AlamarBlue™ Cell Viability Reagent (cat. No. DAL1100, Thermo Fisher Scientific, USA) according to the manufacturer’s instructions. Briefly, the reagent was added directly to the culture medium in each well at a 1:10 ratio (10 µL reagent per 90 µL medium), and the plates were incubated at 37 °C for 24 h.

Each biological sample was analysed in three technical replicate wells. Cell viability was expressed as a percentage of the control group, which was set to 100%. The results were summarised as mean ± SD and used as a pilot cytotoxicity/viability screen to verify that the tested irisin concentrations did not reduce APc viability.

### Total RNA isolation

Total RNA was isolated using Extrazol reagent (BLIRT, Poland) following the manufacturer’s protocol. RNA concentration (A260) and purity (A260/A280 ratio) were measured using a Tecan Infinite M200 plate reader (Tecan Group Ltd., Switzerland). RNA integrity number (RIN) was assessed with an Agilent Bioanalyzer 2100 (Agilent Technologies, USA); only samples with RIN > 8 were used for RNA high-throughput transcriptome sequencing (RNA-Seq), polymerase chain reaction (PCR), and quantitative PCR (qPCR) analyses.

### Library preparation and RNA-Seq

Global gene expression analysis of the porcine APc was performed for both the irisin-treated and control groups (*n* = 5 per group). Ribosomal RNA was depleted using the Ribo–Zero Gold rRNA Removal Kit (Illumina, USA). RNA fragmentation was achieved by incubating the samples in divalent cation buffers at elevated temperatures, using the NEBNext^®^ Magnesium RNA Fragmentation Module (New England Biolabs, USA). Complementary DNA (cDNA) libraries were prepared using the TruSeq Stranded mRNA Library Prep Kit (Illumina, USA), following the manufacturer’s protocol. In brief, mRNA was selectively transcribed into cDNA using poly(dT) oligonucleotides after RNA fragmentation. The resulting double-stranded cDNA underwent end-repair, A-tailing, and adaptor ligation. Enrichment of the cDNA libraries was achieved through PCR amplification. Library quality and quantity were assessed using the Agilent 2100 Bioanalyzer and the High Sensitivity DNA Chip. Paired-end sequencing was subsequently performed on the Illumina’s NovaSeq 6000 platform, generating 2 × 150 nt reads with a targeted minimum sequencing depth of 40 million reads per sample.

### Bioinformatic analysis

Transcriptomic analysis of DEGs, lncRNAs, and AS events was performed as described previously^36,37^. Data visualisation was conducted using SR: plot online tools (https://www.bioinformatics.com.cn/en).

### Differentially expressed transcripts

Raw reads were cleaned using Cutadapt v1.9^38^ to remove adapters, low-quality reads (QPhred score ≤ 20), and reads with > 5% unknown nucleotides. Read quality was assessed with FastQC v0.11.9^39^. Clean reads were aligned to the *Sus scrofa domestica* genome (v.107, Ensembl database) using HISAT2 v2.0.4. Transcript assembly was performed with StringTie v1.3.4d and merged using gffcompare v0.9.8^40,41^. Expression levels of all transcripts were estimated with StringTie and Ballgown v2.30.0^42^. StringTie scripts were also used to calculate fragments per kilobase of transcript per million fragments mapped values for both mRNAs and lncRNAs. Differential expression analysis of mRNAs and lncRNAs between the control and irisin-treated groups was conducted using DESeq2 v1.34.0^43^. Transcripts with an adjusted p-value < 0.05 and absolute log_2_ fold change |log_2_FC| ≥ 0.56 were considered significant.

### Functional annotation of target genes

Gene set enrichment analysis (GSEA) was performed with GSEA software v4.1.0^44^ using the Molecular Signatures Database. Enrichment ontology and pathway analyses were conducted using SR: plot software^45^, incorporating data from the Kyoto Encyclopedia of Genes and Genomes (KEGG)^46–49^ and Gene Ontology (GO)^50,51^ databases. The gene expression matrix and ranked gene lists were processed using the Signal2Noise normalisation method. KEGG pathways and GO terms with p-values < 0.05 were deemed statistically significant.

### lncRNA identification and functional analysis

To identify novel lncRNAs, transcripts overlapping with known mRNAs, annotated lncRNAs, or shorter than 200 bp were excluded. The Coding Potential Calculator (CPC, v0.9-r2)^52^ and the Coding-Non-Coding Index (CNCI, v2.0)^53^ were used to predict the coding potential of transcripts. Transcripts with CPC score < 0.5 and CNCI score < 0 were retained and classified as novel lncRNAs.

Reconstruction and classification of novel transcripts were performed using StringTie and HISAT2, followed by alignment to the reference genome with gffcompare^40,41,54^. Transcripts that neither matched nor overlapped with annotated genes were designated as novel lncRNAs. These novel lncRNAs were further categorised into five classes based on the StringTie-generated class^41^ codes: (i) Intronic: a transfrag entirely within a reference intron; (j) Potentially novel isoform or fragment: sharing at least one splice junction with a reference transcript; (o) Generic exonic overlap: overlapping a reference transcript exon; (u) Intergenic: unknown transcript located in intergenic regions; (x) Antisense: exonic overlap with a reference transcript on the opposite strand. Python script was employed to select regions 100,000 bp upstream and downstream of coding genes to identify potential *cis* interactions of lncRNAs with neighbouring genes. Differential expression of lncRNA sequences between the control and irisin-treated groups was assessed using DESeq2 software^43^. Functional analysis of the target genes associated with these lncRNAs was conducted using GO enrichment analysis and KEGG pathway analysis^47–49^. Statistically significant results were defined by a p-value < 0.05.

### AS event analysis

Differential AS (DAS) analysis was conducted using rMATS v4.1.1^55^ on 120 bp trimmed reads. AS events were quantified as percent spliced-in values across various splicing sites at intron-exon junctions. AS events with a false discovery rate (FDR), q-value < 0.05, and an absolute inclusion level difference (|ΔPSI|) > 0.1 were considered significant. DAS types included alternative 3′ splice site (A3SS), 5′ splice site (A5SS), mutually exclusive exons (MXE), retention intron (RI), and skipping exon (SE).

### qPCR validations of DEGs and lncRNA

The same RNA samples used for RNA-Seq were utilised to synthesise cDNA. QPCR validation of selected DEGs and differentially expressed long noncoding RNAs (DELs) was performed on an AriaMX Real-Time PCR System (Agilent Technologies, USA) following Smolinska et al.^56^. Reverse transcription was conducted using the TranScriba Kit (A&A Biotechnology, Gdańsk, Poland) according to the manufacturer’s protocol. Primer sequences and qPCR conditions are listed in Supplementary Table 1. 18 S ribosomal RNA (*18S rRNA*) and β-actin (*ACTB*), constitutively expressed genes, were used as references. Negative controls contained water instead of cDNA. All reactions were run in duplicates, and their specificity was verified via melting curve analyses. Gene expression was calculated using the comparative cycle threshold method (2^−ΔΔCT^) according to Livak and Schmittgen^57^ and normalised to the geometric mean of reference Ct values. Data normality was confirmed with the Shapiro–Wilk test (*p* > 0.05), and statistical comparisons were made using the Student’s t-test (*p* < 0.05). Statistical analyses were conducted in Statistica software (Statsoft Inc., USA).

### PCR validation of AS events

Selected AS events were validated by PCR using a Labcycler 48s (Syngen Biotech, Poland) with StartWarm HS-PCR Mix (A&A Biotechnology, Gdańsk, Poland). Primers and PCR conditions are detailed in Supplementary Table 1. Amplicons were resolved on 1.5% agarose gels stained with Midori Green Advance (Nippon Genetics Europe, Düren, Germany).

## Results

### Plasma irisin concentrations in pregnant gilts

**Fig. 1:**
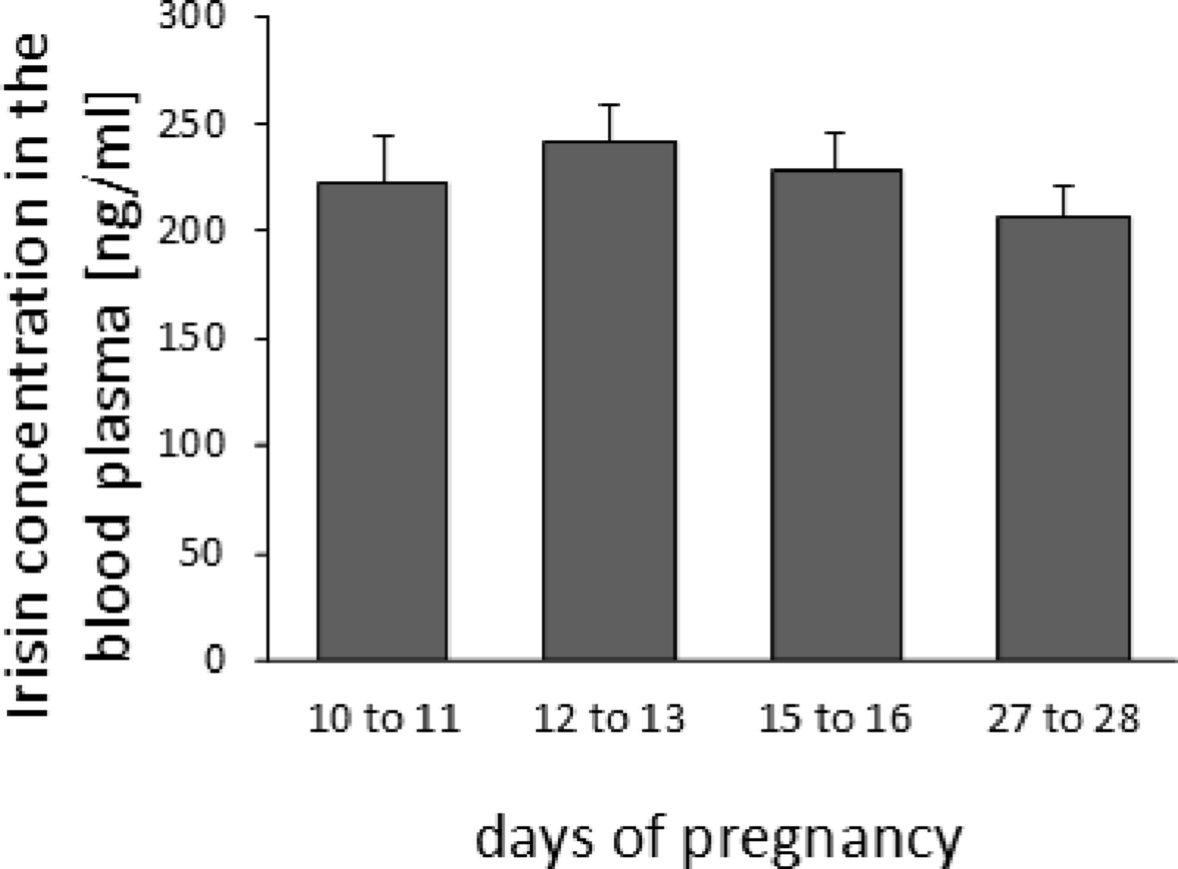
Circulating irisin concentrations in pregnant gilts at selected stages of gestation. Plasma irisin levels were measured by ELISA in gilts on days 10–11, 12–13, 15–16, and 27–28 of pregnancy (*n* = 5 per group). Data are presented as mean ± SD.

Plasma irisin concentrations in pregnant gilts remained within a comparable range across the analysed gestational windows (Fig. 1). Mean ± SD values were 222.31 ± 21.59 ng/mL on days 10–11, 241.77 ± 17.63 ng/mL on days 12–13, 227.96 ± 17.61 ng/mL on days 15–16, and 206.54 ± 13.89 ng/mL on days 27–28 of pregnancy. Individual values ranged from 143.03 to 302.85 ng/mL, with the highest point observed during days 12–13 of pregnancy. The overall mean ± SD value for all samples was 224.65 ± 9.38 ng/mL; therefore, 300 ng/mL, representing the upper physiological range, was selected for the in vitro experiments.

### Dose-response viability assay

To verify whether the irisin concentrations used in the in vitro experiment affected APc viability, a dose-response assay was performed using the AlamarBlue method in cells isolated from gilts on days 2–3, 10–12, 14–16, and 17–19 of the oestrous cycle (Table 1). After 24 h exposure to 100, 200, or 300 ng/mL irisin, no reduction in cell viability indicative of cytotoxicity was observed in any of the analysed periods. These findings showed that irisin at 300 ng/mL, the concentration selected for the in vitro experiments, did not exert cytotoxic effects on porcine APc cultured in vitro.

**Table 1:** Viability of porcine anterior pituitary cells (APc) after 24 h in vitro exposure to irisin at concentrations of 100, 200, and 300 ng/mL, assessed by the alamarBlue assay. Cells were isolated from gilts on days 2–3, 10–12, 14–16, and 17–19 of the oestrous cycle. Results are presented as mean ± SD and expressed as percentage of the control group (control = 100%).

Days of oestrous cycle		Irisin 100 ng/mL	Irisin 200 ng/mL	Irisin 300 ng/mL
2–3	Mean	113.902	111.1537	112.6614
	± SD	9.025063	12.25818	11.40531
10–12	Mean	108.808	117.7734	115.4086
	± SD	9.830833	26.5711	13.70581
14–16	Mean	100.1809	96.93898	101.1035
	± SD	0.974237	2.022269	3.89572
17–19	Mean	91.14708	91.05397	99.10109
	± SD	3.650499	5.565606	1.940662

### General results of sequencing

The raw RNA-Seq data were deposited in the European Nucleotide Archive (ENA) database (https://www.ebi.ac.uk/ena) under accession number PRJEB98233. Sequencing generated 568,313,700 raw paired-end reads, with an average of 56.8 million reads per sample (Supplementary Table 2). Among these, 334,324,797 reads (average mapping rate: 66.2%) aligned successfully to the *Sus scrofa* reference genome (v107). On average, 58.37% of reads mapped to exonic regions, 37.65% to intronic regions, and 3.98% to intergenic regions. Detailed mapping statistics are provided in Supplementary Table 2.

### DEGs and functional annotations

**Fig. 2:**
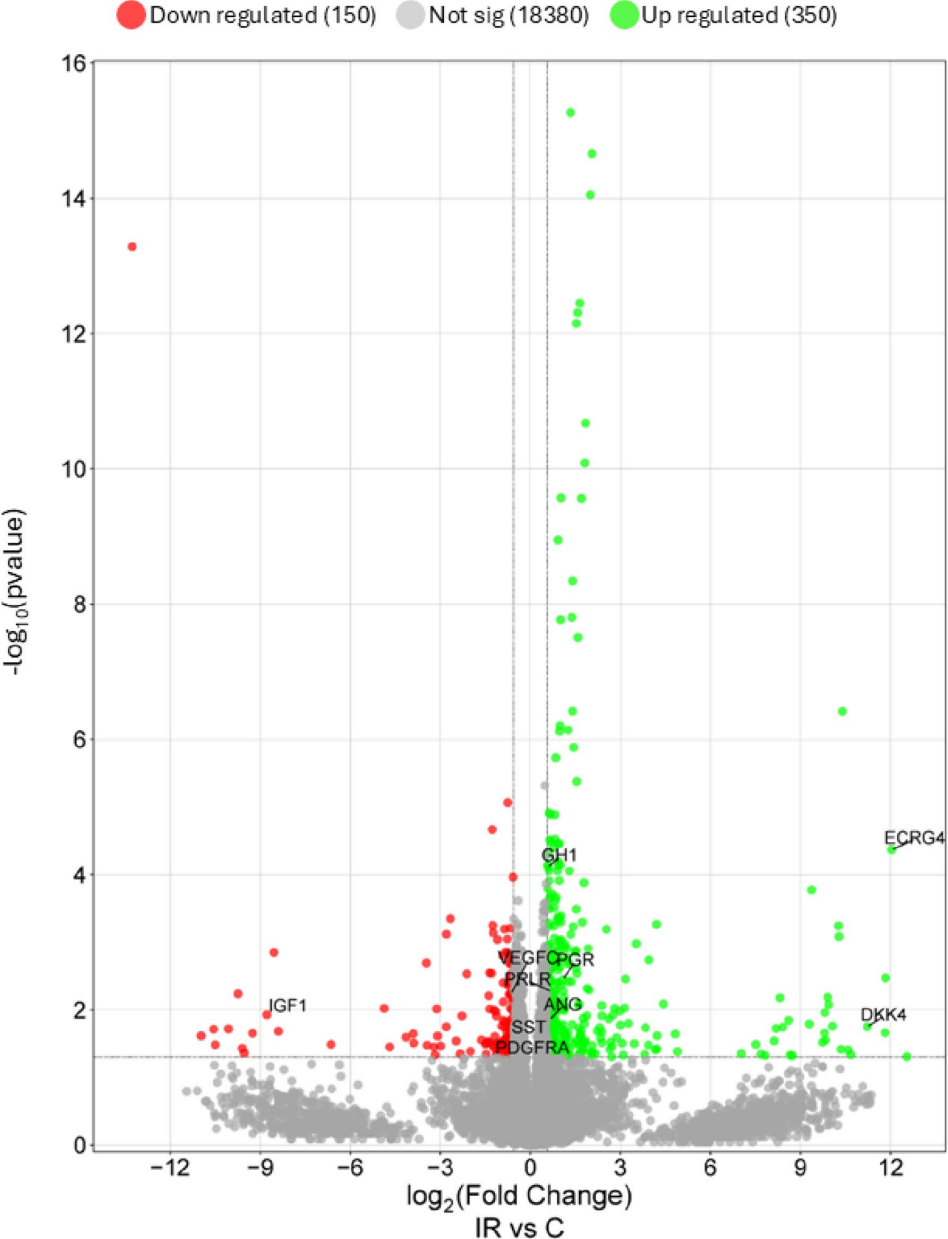
Visualisation of genes with expression altered by irisin. Volcano plot of differentially expressed genes (DEGs) in irisin-treated (IR) anterior pituitary cells versus controls (C). The X-axis shows log_2_ fold change (log_2_FC); the Y-axis shows the negative decimal logarithm of p-values (− log_10_(p-value)). The horizontal line marks the significance threshold (*p* = 0.05; − log_10_(p-value) = 1.3). Vertical lines denote the fold-change cut-off (log_2_FC > |0.56|). Points are coloured by significance and direction of change. *IGF1* insulin-like growth factor 1, *ANG* angiogenin, *DKK4* dickkopf-related protein 4, *ECRG4* oesophageal cancer-related gene 4, *GH1* growth hormone 1, *PDGFRA* platelet-derived growth factor receptor alpha, *PGR* progesterone receptor, *PRLR* prolactin receptor, *SST* somatostatin, *VEGFC* vascular endothelial growth factor C.

**Fig. 3:**
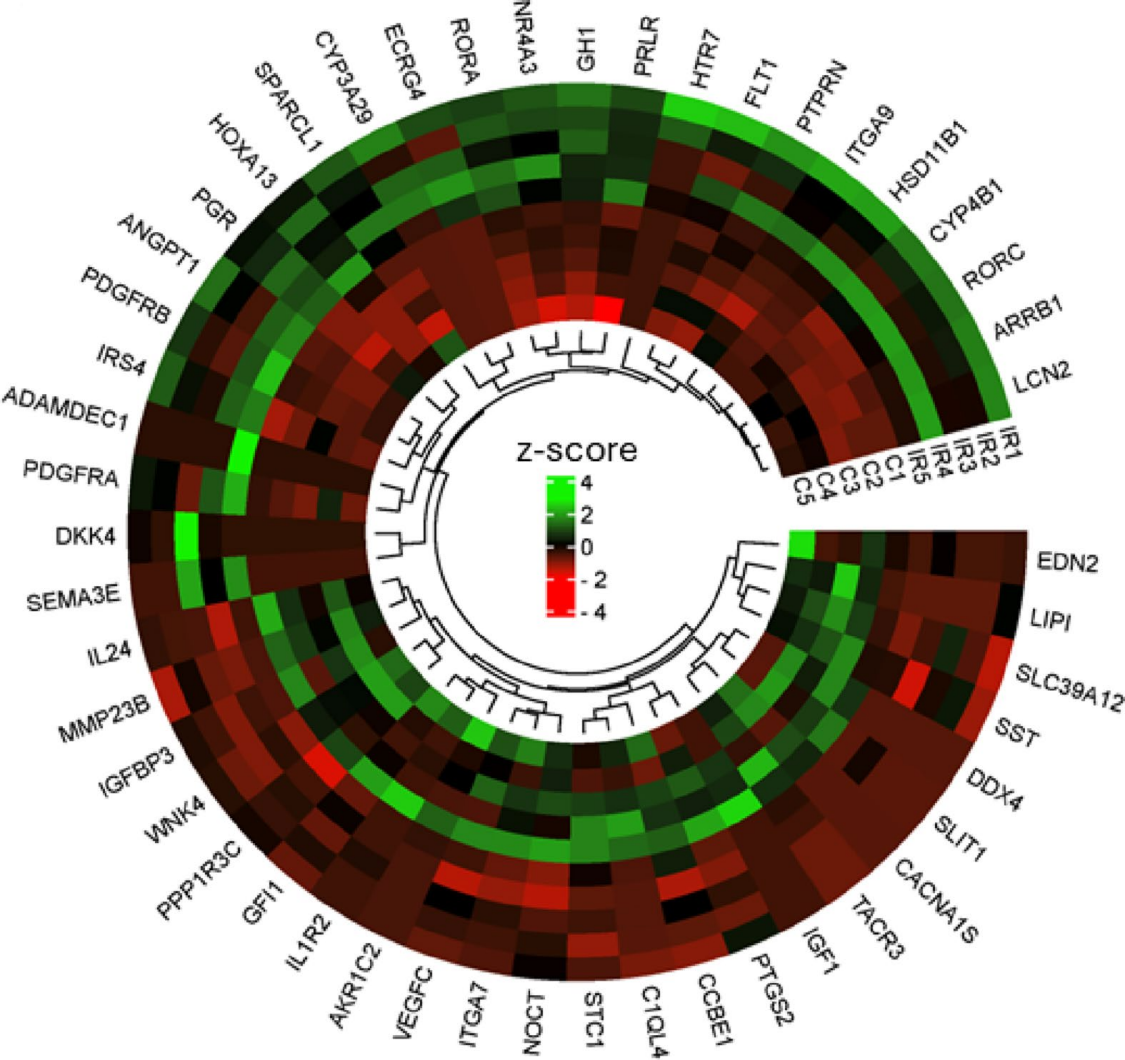
Hierarchical clustering heatmap of selected irisin-regulated genes. Heatmap showing normalised expression (Z‑score; red–green scale) of selected differentially expressed genes (DEGs) across biological replicates of control (C1–C5) and irisin‑treated (IR1–IR5) anterior pituitary cells. Colour intensity reflects relative expression level after normalisation.

**Fig. 4:**
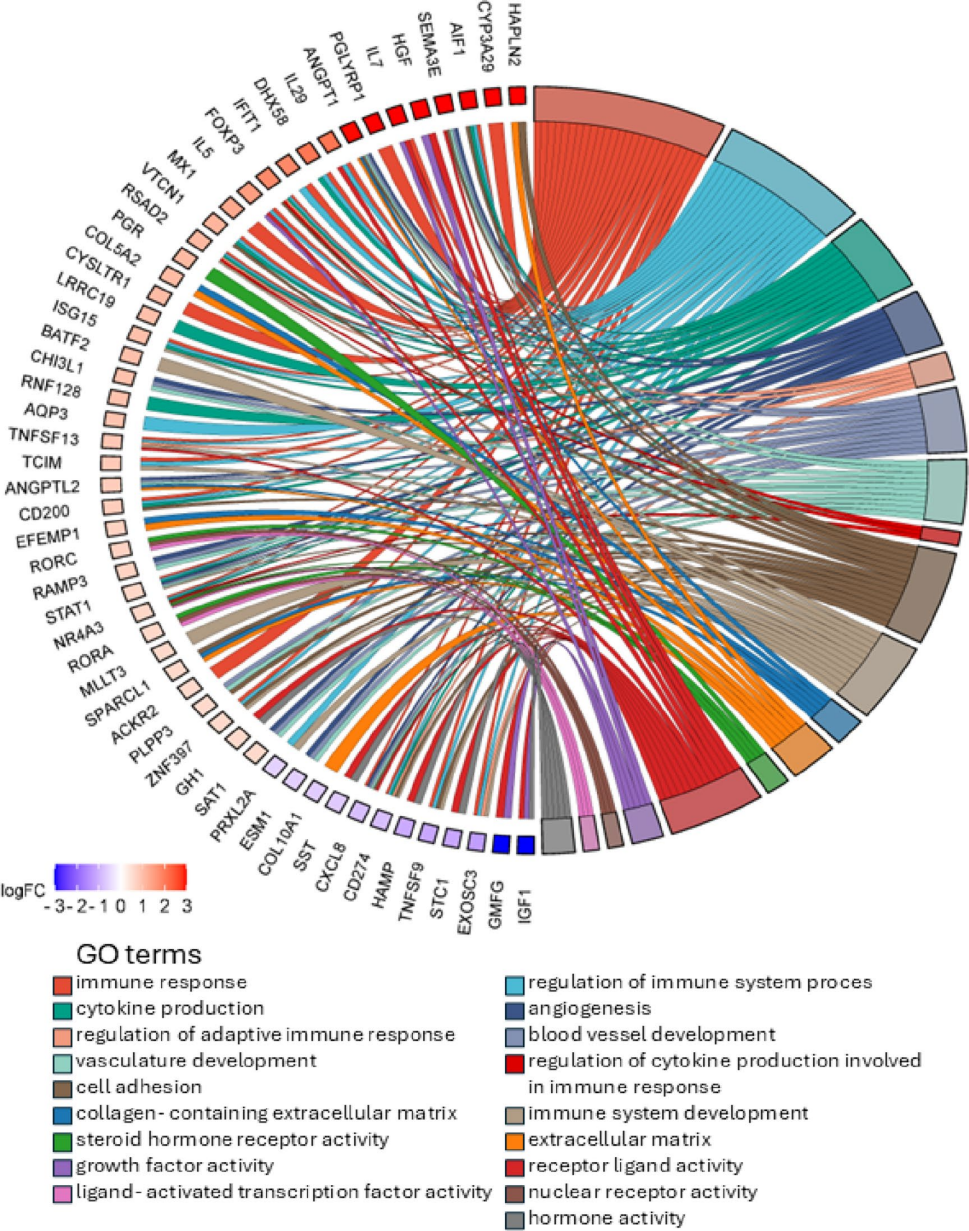
Gene Ontology (GO) enrichment of differentially expressed genes (DEGs) altered by irisin. Circos plot of selected GO terms significantly enriched among DEGs identified in irisin-treated anterior pituitary cells (*p* < 0.05; log_2_FC > |0.56|). Connecting line colour represents a distinct GO term. Bars adjacent to gene symbols indicate the proportion of upregulated (red) and downregulated (blue) genes associated with each term.

**Fig. 5:**
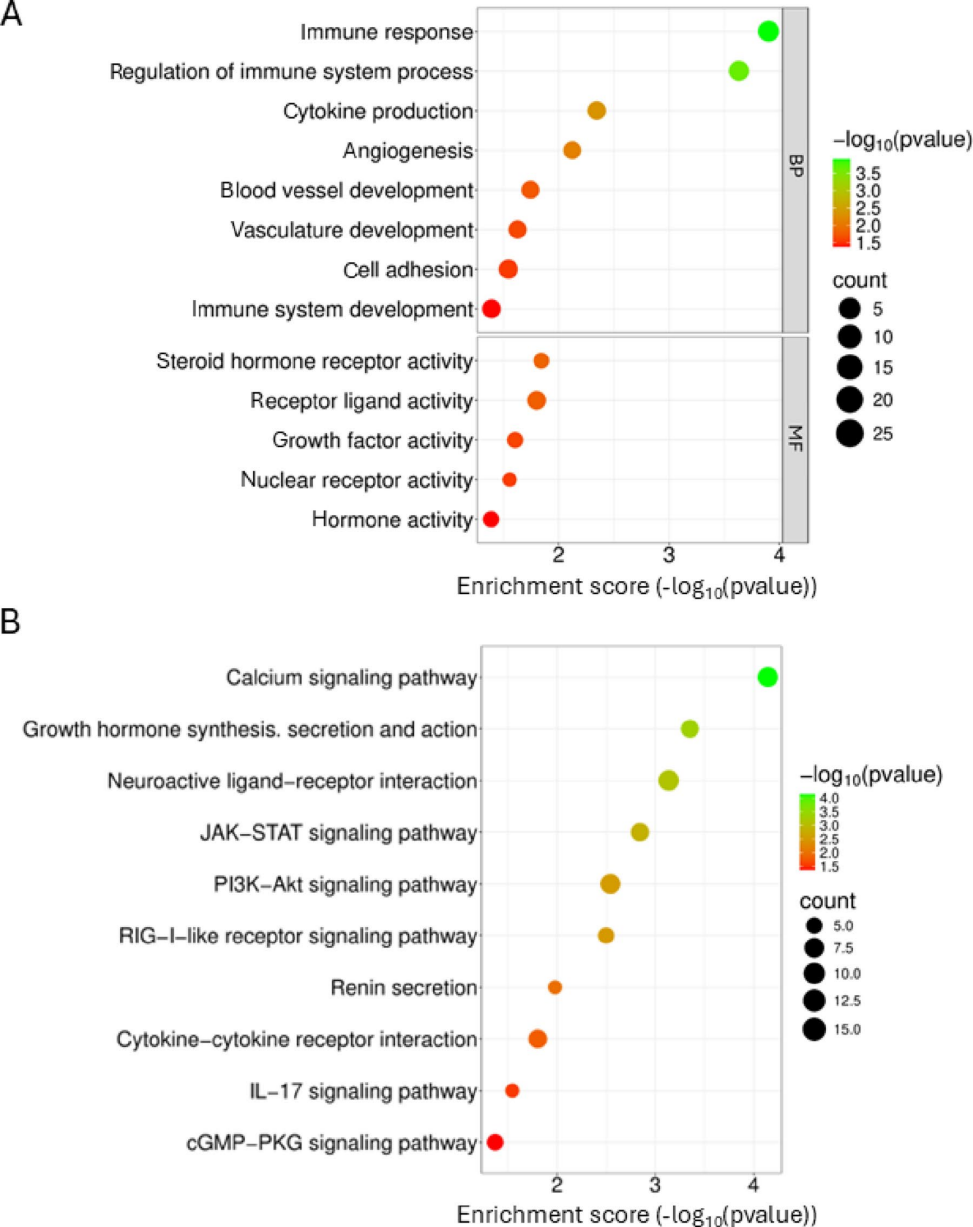
Gene Ontology (GO) terms and Kyoto Encyclopedia of Genes and Genomes (KEGG) pathways enriched in irisin-regulated differentially expressed genes (DEGs). (**A**) GO enrichment summary for DEGs in Biological Process (BP) and Molecular Function (MF) categories. (**B**) KEGG pathway enrichment analysis for the same DEG set. In both panels, the vertical axis lists enriched GO terms or KEGG pathways, and the horizontal axis shows enrichment score (calculated as the negative decimal logarithm of the pathway’s p-value (− log_10_(p-value)). Dot size reflects the number of DEGs annotated to each term/pathway, and dot colour denotes gene regulation (upregulated—green; downregulated—red).

RNA-Seq identified 18,887 genes, of which 500 met the thresholds of |log_2_FC| > 0.56 and adjusted p-value < 0.05. Among these, 350 genes were upregulated and 150 genes downregulated in response to irisin (Fig. 2). The log_2_FC values of DEGs ranged from − 13.27 (Zinc Finger HIT-Type Containing 2 (*ZNHIT2*)) to 12.54 (*ENSSSCG00000034115*) (Supplementary Table 3). The expression patterns of representative DEGs are visualised in the heatmap (Fig. 3). GO analysis revealed 180 significantly enriched terms (adjusted *p* < 0.05), including 159 biological processes (BP), 4 cellular components (CC), and 17 molecular functions (MF) (Figs. 4 and 5A; Supplementary Table 4). Within BP, the most interesting terms included ‘immune response’ (GO:0006955), ‘angiogenesis’ (GO:0001525), ‘cell adhesion’ (GO:0007155), and ‘cytokine production’ (GO:0001816). For CC, DEGs were predominantly linked to the ‘intrinsic component of plasma membrane’ (GO:0031226), ‘integral component of plasma membrane’ (GO:0005887), ‘extracellular matrix’ (GO:0031012), and ‘collagen-containing extracellular matrix’ (GO:0062023). In the MF category, the most significant enrichments included ‘receptor ligand activity’ (GO:0048018), ‘hormone activity’ (GO:0005179), ‘growth factor activity’ (GO:0008083), and ‘steroid hormone receptor activity’ (GO:0003707). These enrichments are visualised in Fig. 5A. KEGG pathway analysis identified 33 significantly enriched signalling pathways (adjusted *p* < 0.05), with the most prominent being ‘neuroactive ligand–receptor interaction’ (KEGG:04080). Other highly represented pathways included ‘growth hormone synthesis, secretion, and action’ (KEGG:04935), ‘calcium signaling pathway’ (KEGG:04020), ‘phosphoinositide 3-kinase/protein kinase B (PI3K–Akt) signaling pathway’ (KEGG:04151), and ‘Janus kinase—signal transducer and activator of transcription (JAK–STAT) signalling’ pathway (KEGG:04630). Detailed pathway annotations are available in Supplementary Tables 5 and visualised in Figs. 5B and Supplementary Figs. 1 and 2.

### LncRNA identification and functional annotations

**Fig. 6:**
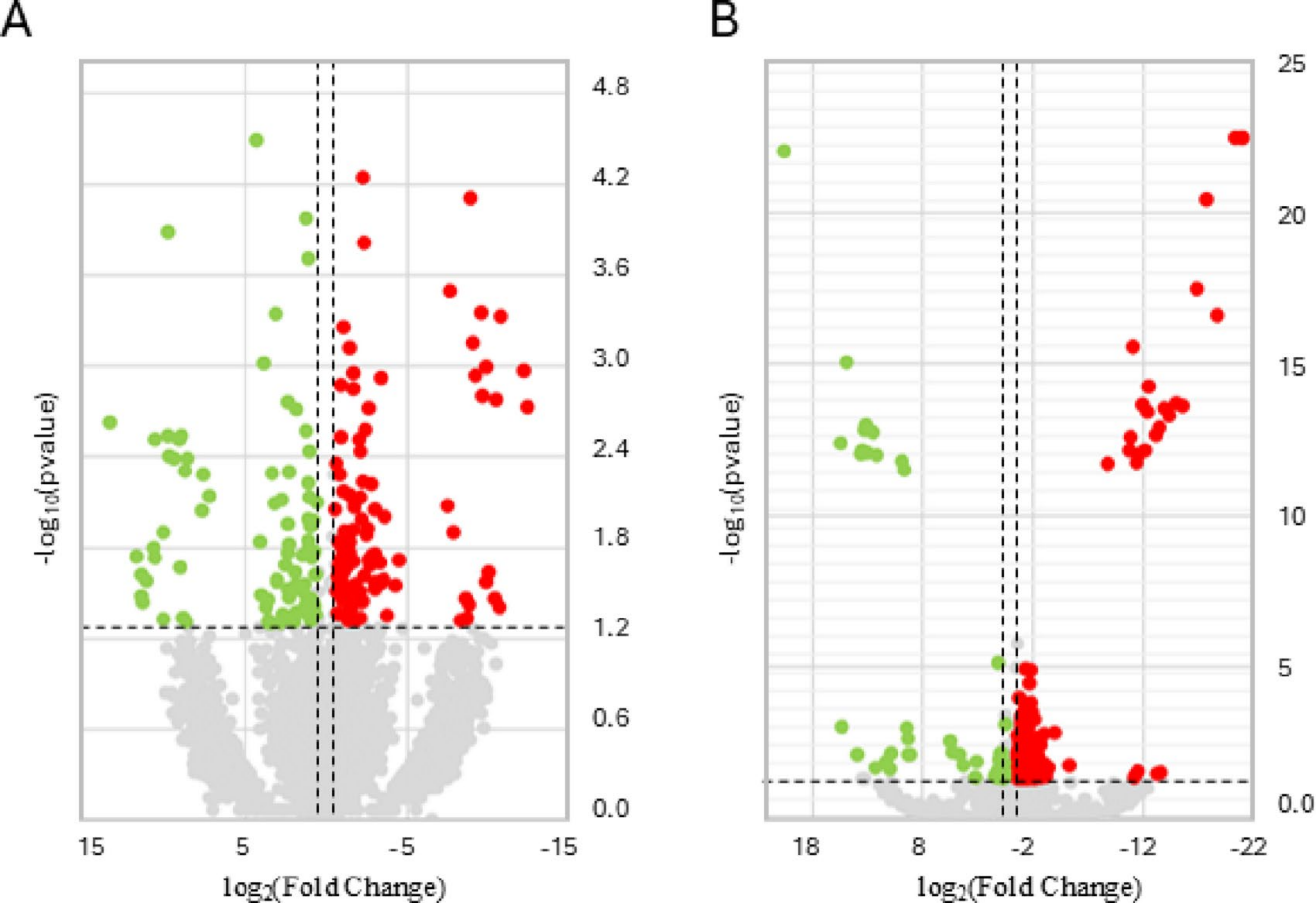
Visualisation of long non-coding RNA (lncRNA) regulated by irisin. (**A**) lncRNAs annotated in Ensembl database. (**B**) Novel lncRNA candidates predicted in this study. Volcano plots display differentially expressed lncRNAs (DELs) between irisin-treated (IR) and control (C) anterior pituitary cells. The X-axis shows log_2_ fold change (log_2_FC); the Y-axis shows the negative decimal logarithm of the p-values (− log_10_(p-value)). The horizontal line marks the significance threshold (*p* = 0.05; − log_10_(p-value) = 1.3). Vertical lines represent the fold change cut-offs (log_2_FC > |0.56|). Point colours indicate significance and direction of the change.

**Fig. 7:**
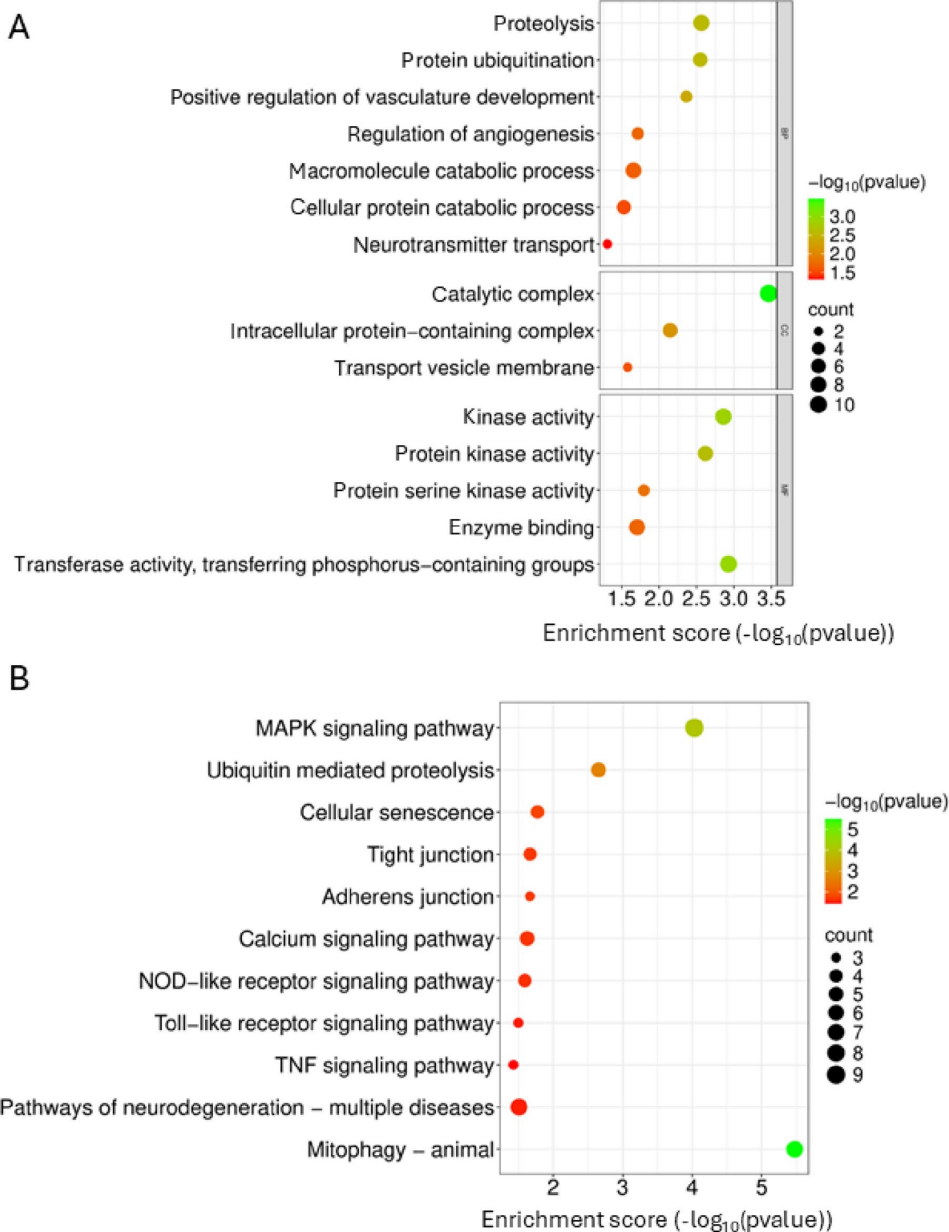
Gene Ontology (GO) and Kyoto Encyclopedia of Genes and Genomes (KEGG) pathways enrichment analyses of predicted target genes of differentially expressed long non-coding RNAs (DELs). (**A**) Selected GO terms enriched among predicted cis-target genes of DELs. (**B**) KEGG pathway enrichment for the same gene set. The vertical axis lists enriched GO terms or KEGG pathways, the horizontal axis shows enrichment score (calculated as the negative decimal logarithm of the pathway’s p-value (− log_10_(p-value)). Dot size reflects the number of DELs annotated to each term/pathway, and dot colour indicates regulation of associated genes (upregulated—green; downregulated—red).

A total of 15,772 lncRNA candidates were identified, of which 747 were differentially expressed (*FDR < 0.05*, log_2_|FC| > 0.56), hereafter referred to as DELs. Among these, 349 were annotated in the ENSEMBL database (Fig. 6A), while 398 were classified as novel lncRNAs (Fig. 6B). In the irisin-treated group, 314 lncRNAs were upregulated and 433 were downregulated, with log_2_FC values ranging from − 20.97 to 20.56 (Supplementary Table 6). We identified 178 potential DEL-DEG interaction events based on genomic colocalisation, with 143 within 100 kb, 29 within 10 kb, and 6 within 1 kb. Because no strongly correlated DEL–DEG pairs were detected (r^2^ > 0.9; *FDR < 0.05*; log_2_|FC| > 0.56), the analysis of genes located within the predefined cis-association window was included only as a preliminary and indicative exploration of potentially relevant biological processes. GO analysis revealed 82 significantly enriched terms across three categories (Fig. 7A, Supplementary Table 7). Among these, 40 BP terms were enriched, with ‘proteolysis’ (GO:0006508) being most prominent. Within CC, 22 terms were significant, including ‘catalytic complex’ (GO:1902494) associated with 11 genes. For MF, 20 enriched terms were identified, with the strongest enrichment for ‘transferase activity and transferring phosphorus-containing groups’ (GO:0016772). KEGG pathway analysis revealed 32 significantly enriched pathways for DEL-associated genes (Fig. 7B, Supplementary Table 7). The most enriched pathway was the ‘mitogen-activated protein kinase (MAPK) signalling pathway’ (ssc04010; 9 genes), followed by ‘pathways of neurodegeneration – multiple diseases’ (ssc05022; 7 genes) and ‘mitophagy—animal’ (ssc04137; 7 genes).

### Alternative splicing event analysis

**Fig. 8:**
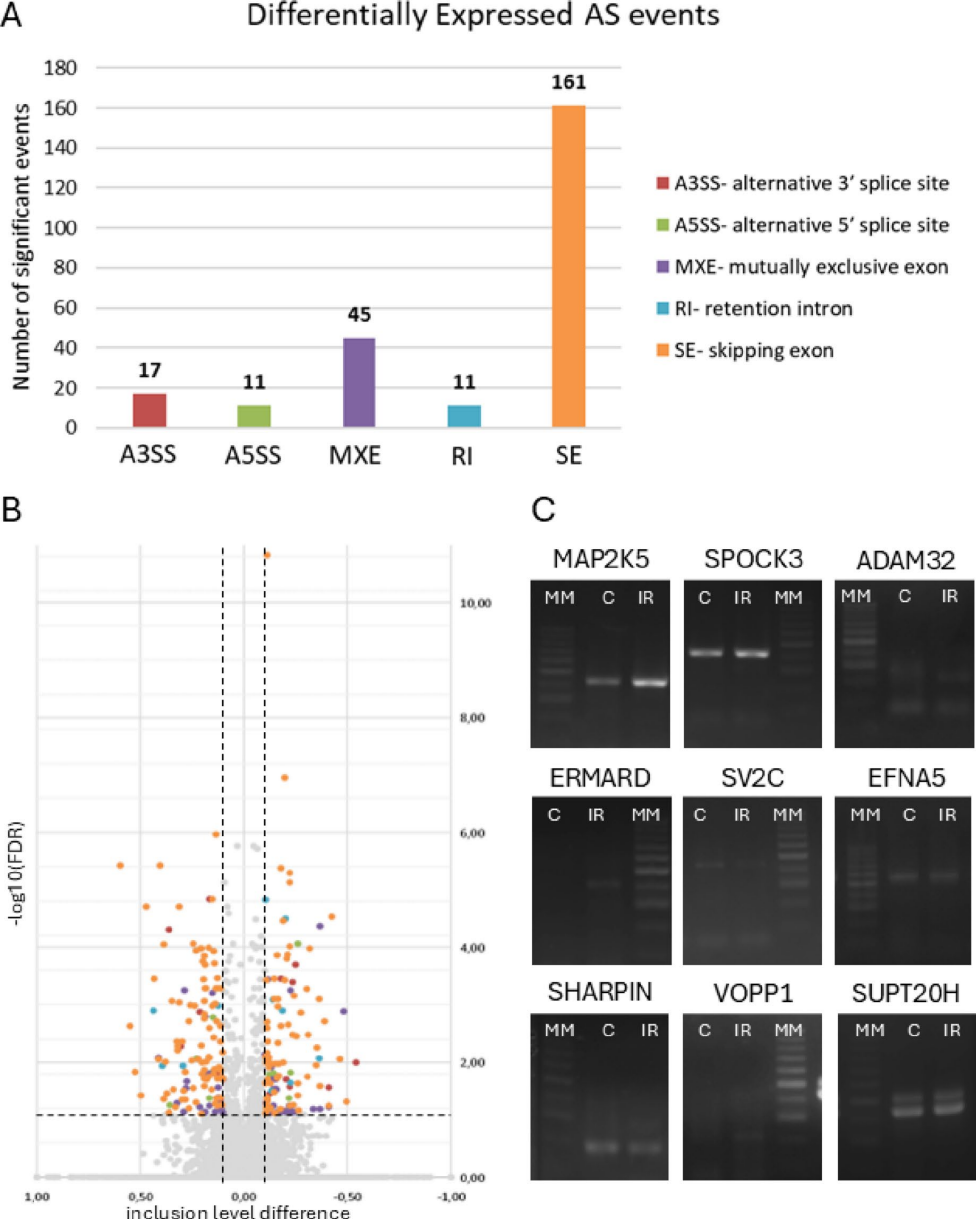
Differentially alternative splicing (DAS) events regulated by irisin. (**A**) Distribution of DAS events by category: alternative 3′ splice site (A3SS), alternative 5′ splice site (A5SS), mutually exclusive exons (MXE), retained intron (RI), and skipped exon (SE). (**B**) Volcano plot of DAS events. The X‑axis shows inclusion level difference (ΔPSI) between irisin-treated (IR) and control (C) groups; the Y‑axis shows the negative decimal logarithm of the p‑value (− log_10_(p-value)). Horizontal and vertical reference lines denote statistical significance threshold (*p* = 0.05; − log_10_(p-value) = 1.3) and the fold change cut-offs (log_2_FC > |0.56|). Points are coloured by DAS category. (**C**) Validation of selected DAS events by PCR/gel electrophoresis, illustrating exon inclusion versus skipping in representative IR and C samples.

In total, 18,365 AS events were detected, of which 245 were significantly different between the irisin-treated and control APc. These DASs were classified into five categories: 17 were classified as A3SS, 11 as A5SS, 45 as MXEs, 11 as RIs, and 161 as SEs (Fig. 8A,B). The inclusion level differences for these events ranged from − 0.542 to 0.596. Comprehensive details of the DAS analysis are presented in Supplementary File 8.

### Validation of RNA-Seq results

**Fig. 9:**
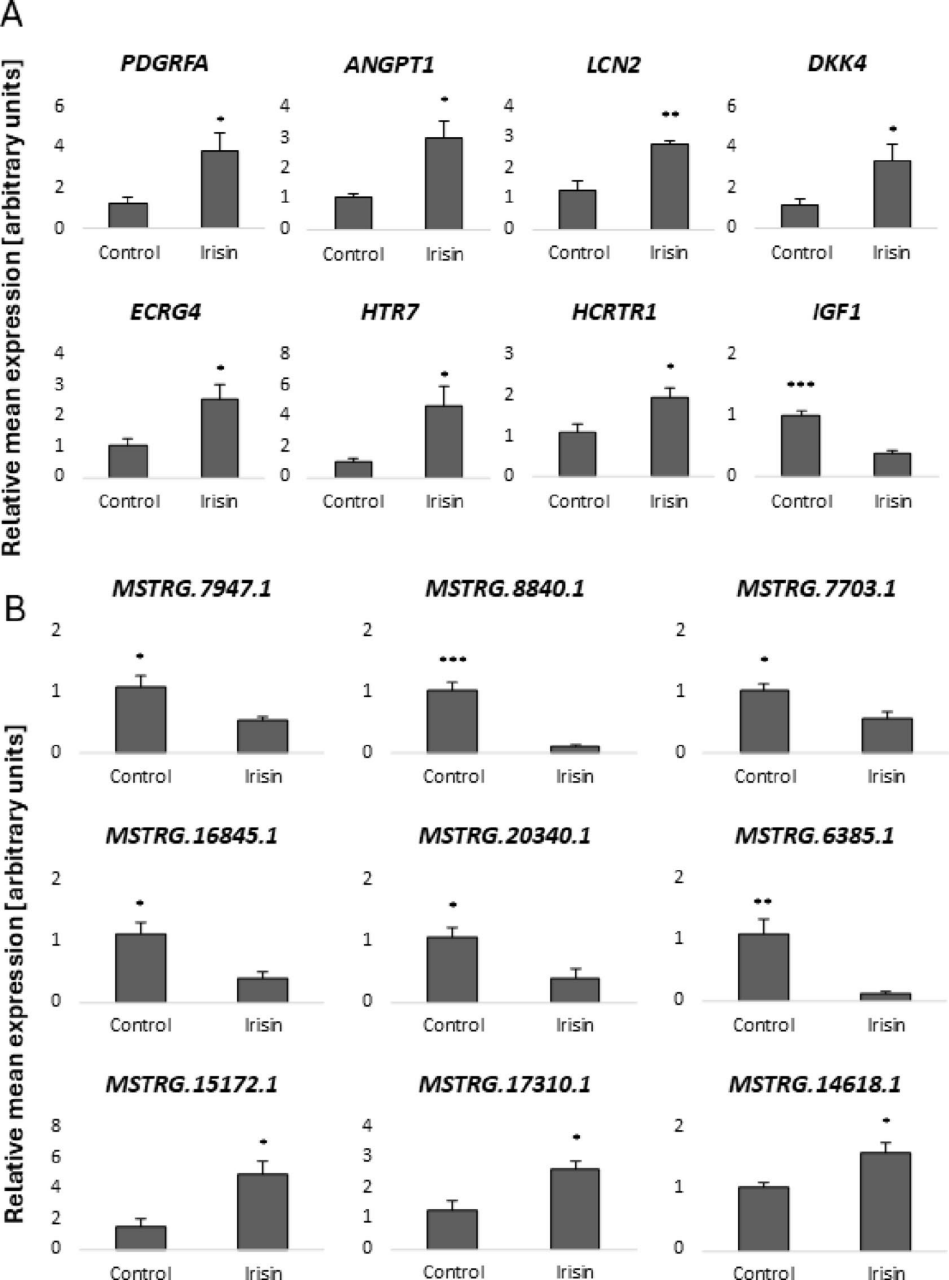
Quantitative real‑time PCR (qPCR) validation of high-throughput transcriptome sequencing (RNA‑Seq) results for irisin-regulated differentially expressed genes (DEGs) and long non-coding RNAs (lncRNAs). (**A**) Validation of selected DEGs. (**B**) Validation of selected lncRNAs. Genes validated include *PDGFRA*, *ANGPT1*, *LCN2*, *DKK4*, *ECRG4*, *HTR7*, *HCRTR1*, and *IGF1*. Validated lncRNAs include *MSTRG.7947.1*, *MSTRG.8840.1*, *MSTRG.7703.1*, *MSTRG.16845.1*, *MSTRG.20340.1*, *MSTRG.6385.1*, *MSTRG.15172.1*, *MSTRG.17310.1*, and *MSTRG.14618.1*. Expression values were normalised to 18 S rRNA and ACTB. Statistical significance threshold: *p* < 0.05.

Eight DEGs: platelet-derived growth factor receptor alpha (*PDGFRA*), angiopoietin-1 (*ANGPT1*), lipocalin-2 (*LCN2*), dickkopf-related protein 4 (*DKK4*), oesophageal cancer-related gene 4 (*ECRG4*), 5-hydroxytryptamine (serotonin) receptor 7 (*HTR7*), hypocretin receptor 1 (*HCRTR1*), insulin-like growth factor 1 (*IGF1*), along with nine lncRNAs: *MSTRG.7947.1*, *MSTRG.8840.1*, *MSTRG.7703.1*, *MSTRG.16845.1*, *MSTRG.20340.1*, *MSTRG.6385.1*, *MSTRG.15172.1*, *MSTRG.17310.1*, *MSTRG.14618.1* were selected for qPCR validation. Additionally, nine genes showing DAS events were tested: activated protein kinase kinase 5 (*MAP2K5*), sparc/osteonectin, cwcv, and kazal-like domains proteoglycan 3 (*SPOCK3*), ADAM metallopeptidase domain 32 (*ADAM32*), early response to malaria and related diseases (*ERMARD*), synaptic vesicle glycoprotein 2c (*SV2C*), ephrin-a5 (*EFNA5*), shank-associated rh domain interacting protein (*SHARPIN*), vesicular, overexpressed in cancer, prosurvival protein 1 (*VOPP1*), spt20 homolog, saga complex component (*SUPT20H*). QPCR results confirmed the RNA-Seq findings for DEGs and DELs (Fig. 9), while PCR validation confirmed DAS patterns (Fig. 8C). These results support the accuracy and reliability of the RNA-Seq and bioinformatic analyses used in this study.

## Discussion

The pituitary gland, a central endocrine organ, plays a crucial role in regulating reproductive function through complex hormonal signalling pathways^58^. Pituitary-derived hormones are essential for gametogenesis, steroidogenesis, and overall reproductive processes, as well as numerous other physiological functions^18^. The secretory activity of the pituitary is, in turn, tightly controlled by feedback mechanisms and biologically active substances, such as cytokines or adipokines, that reach the gland^4,5,59^. Among these factors, irisin appears to act as a potential modulator of pituitary activity, particularly in a reproductive context. In this study, we investigated the effect of irisin on the global transcriptomic profile of porcine APc isolated from gilts on days 15–16 of pregnancy using RNA-Seq. Our analysis revealed 500 DEGs, 747 DELs, and 245 significant AS events. Functional enrichment highlighted 180 GO terms and 33 KEGG pathways associated with endocrine regulation, angiogenesis, calcium signalling, and extracellular matrix remodelling. To our knowledge, this is the first study to comprehensively characterise the effects of irisin on the transcriptomic profile of the anterior pituitary during the peri-implantation period. Although this primary in vitro culture model does not fully reproduce the complex endocrine milieu of early pregnancy throughout the entire experimental period, it remains one of the most informative and widely accepted approaches for analysing direct cellular responses to defined stimuli under controlled conditions. Importantly, the cells were isolated from gilts at a defined physiological stage, and their prior in vivo hormonal exposure is likely to influence baseline molecular responsiveness, despite the absence of the complete pregnancy environment ex vivo. Therefore, the present dataset should be considered hypothesis-generating and requires further validation at the protein and functional levels to confirm the biological relevance of the observed transcriptomic changes.

The physiological relevance of the irisin concentration used in this study is supported both by our plasma measurements and by previously published data obtained in humans and other animal models. In the analysed gilts, circulating irisin remained within a comparable range across the examined stages of pregnancy, with an overall mean value of 224.65 ± 9.38 ng/mL and individual values approaching 300 ng/mL. Accordingly, 300 ng/mL was selected as an upper-range physiologically relevant concentration rather than an arbitrarily chosen pharmacological dose. Notably, higher serum irisin concentrations have been reported in human pregnancy, including median values of 757 ng/mL in the second trimester^24^, and 1300 ± 200 ng/mL^25^, while Garcés et al. demonstrated sustained elevation throughout pregnancy compared with the non-pregnant state^3^. In dairy cattle, circulating FNDC5/irisin levels also vary with physiological status, ranging from 4.55 ± 0.52 to 11.44 ± 1.14 ng/mL during the peripartum period, with the highest values observed at 1 week postpartum, i.e., during the period of pronounced negative energy balance^60^. Importantly, irisin has been shown to exert direct biological effects in bovine in vitro models, including gonadotroph cultures, where it modulates LH and FSH secretion^30^, supporting its biological activity in large domestic species. In our pilot dose-response assay, irisin at 100, 200, and 300 ng/mL did not affect APc viability after 24 h of incubation, indicating that the concentration used for transcriptomic analysis was not cytotoxic. Furthermore, our data suggest that irisin responsiveness may be influenced by the hormonal status of donor animals, consistent with our previous observation that irisin expression in the porcine pituitary varies across the oestrous cycle and early pregnancy^29^. Collectively, these findings indicate that the observed transcriptomic changes are more likely to reflect a biologically relevant, context-dependent response to irisin rather than nonspecific effects associated with cellular stress.

In the present study, irisin treatment was associated with altered expression of several key genes in the porcine pituitary during early gestation, suggesting a potential regulatory role in endocrine adaptation to pregnancy at the transcriptional level. Among the most affected genes were *IGF1*, somatostatin (*SST*), and growth hormone 1 (*GH1*), which are functionally linked to enriched GO terms such as ‘hormone activity’ (GO:0005179), ‘receptor regulator activity’ (GO:0030545), and ‘receptor ligand activity’ (GO:0048018). Together with additional genes, including calcium voltage-gated channel subunit alpha1 S and F (*CACNA1S*,* CACNA1F*), insulin-like growth factor binding protein 3 (*IGFBP3*), signal transducer and activator of transcription 1 (*STAT1*), insulin receptor substrate 4 (*IRS4*), and SHC adaptor protein 4 (*SHC4*), these DEGs were linked to the KEGG pathway ‘growth hormone synthesis, secretion and action’.

Our findings further indicate a potential involvement of irisin in the regulation of the growth hormone (GH)/IGF1 axis. The pituitary-derived *GH1* gene encodes GH, a key regulator of embryogenesis and systemic development, and an important component of reproductive physiology^61^. The GH/IGF axis also regulates growth and metabolism in reproductive contexts^62^. In the present dataset, irisin upregulated *GH1* expression while downregulating *SST*, a known inhibitor of GH secretion, suggesting a possible transcript-level modulation of GH regulatory pathways. KEGG analyses linked GH1-associated changes to JAK/STAT and PI3K/Akt signalling pathways, indicating that irisin may influence GH-related processes independently of IGF1. It is also possible that irisin may directly influence *GH1* gene expression and potentially affect GH secretion in pituitary cells. However, given the transcriptomic nature of this study, these findings should be interpreted as indicative of potential regulatory mechanisms rather than direct evidence of altered hormone secretion. Considering the complexity of the GH1/IGF1/SST axis and its key role in pituitary regulation of reproductive processes, the observed transcriptomic changes point toward a potential involvement of irisin in modulating this system at the gene expression level.

During early pregnancy, circulating PRL rises steadily, supporting luteal maintenance, upregulation of uterine oestrogen and P4 receptor (PGR) expression, and stimulation of endometrial angiogenesis^63^. PRL also acts locally within the pituitary to induce angiogenic factors, such as vascular endothelial growth factor A (VEGFA), and to stimulate capillary growth around expanding lactotroph clusters^64^. Within this physiological context, our data indicate that irisin may enhance pituitary sensitivity to both PRL and P4 by upregulating the prolactin-receptor isoform 1 (*PRLR1*) and the *PGR* genes, thereby positioning irisin as a potentiator of PRL signalling both directly and indirectly through P4. Mechanistically, PRL–PRLR engagement classically activates the cytosolic tyrosine kinase JAK2^65^. Irisin further amplifies this axis by upregulating interleukins 5 (*IL-5*) and 7 (*IL-7*), which activate JAK1/JAK3, as well as *STAT1* and interferon regulatory factor 9 (*IRF9*), consistent with broader JAK/STAT pathway activation^61^. Given that P4 can act directly on pituitary cells to modulate gonadotrophin secretion in a time-dependent manner^62^ and can stimulate maternal PRL release^66^ irisin-driven upregulation of *PGR* likely strengthens P4’s ability to enhance PRL output, creating a feed-forward environment that supports lactotroph expansion and functional maturation. At the same time, because *PRLR* activation has been linked to lactotroph apoptosis in rodents^67,^ irisin-mediated increases in *PRLR* expression may also contribute to pregnancy-specific pituitary remodelling by balancing lactotroph proliferation and turnover. Collectively, these findings suggest that irisin acts as an integrative modulator of pituitary plasticity in pregnancy by enhancing PRL signalling directly (through *PRLR* upregulation and JAK–STAT engagement) and indirectly (through increased P4 responsiveness *via PGR*), thereby coordinating the structural and secretory adaptations required for gestation and subsequent lactation.

Consistent with these findings, the pituitary undergoes extensive structural remodelling during gestation. Enlargement of the gland and expansion of the lactotroph population are hallmarks of this period^68^. Such changes reflect the gland’s cellular plasticity and its ability to adapt to the endocrine demands of pregnancy^69^. The enrichment of genes assigned to ‘angiogenesis’ (GO:0001525), ‘vasculature development’ (GO:0001944), and ‘cell adhesion’ (GO:0007155) categories suggests a possible association of irisin treatment with remodelling-related transcriptomic patterns. Specifically, irisin upregulated *SEMA3E* gene expression, which encodes a ligand for Plexin–D1, a critical regulator of angiogenic patterning, while downregulating vascular endothelial growth factor C (*VEGFC)* expression, a major driver of lymphangiogenesis^70,71^. Moreover, irisin increased the expression of other pro-angiogenic genes, including angiogenin (*ANG*), angiopoietin 1 and angiopoietin-like 2 (*ANGPT1* and *ANGPTL2*), spermidine/spermine N1-acetyltransferase 1 (*SAT1*), and *STAT1*, which may be consistent with a potential involvement of irisin in vascular remodelling within the pituitary. These observations are in line with reports that irisin may promote endothelial cell proliferation and angiogenesis via the ERK1/2 signalling pathway^72^. Such effects are particularly relevant during pregnancy, when increased vascularisation supports heightened nutrient and hormone demands.

We also observed irisin-driven modulation of genes involved in calcium signalling, a pathway crucial for hormone exocytosis and pituitary electrical activity^73^. Irisin increased expression of *CACNA1F*, encoding an L-type voltage-gated calcium channel subunit, which may be consistent with altered calcium-handling capacity. This finding aligns with studies showing that voltage-gated calcium channels are central to initiating hormone exocytosis in APc^73,74^. Conversely, the decreased expression of *CACNA1S*, another voltage-gated channel gene, suggests that irisin may selectively regulate different calcium channel types, potentially altering the electrical activity patterns in various pituitary cell types. Additionally, irisin upregulated phosphodiesterase 1 C (*PDE1C*), encoding a calcium/calmodulin-regulated phosphodiesterase, and histidine-rich calcium-binding protein (*HRC*), indicating potential modulation of intracellular calcium storage and processing in the endoplasmic reticulum (ER). These findings are consistent with studies showing that irisin promotes calcium reuptake into the ER in vascular smooth muscle cells^75^ and extracellular calcium influx in endothelial cells^76^. Furthermore, increased expression of receptor genes, such as cysteinyl leukotriene receptor 1 (*CYSLTR1*), *PDGFRA*, platelet-derived growth factor receptor beta (*PDGFRB*), platelet-activating factor receptor (*PTAFR*), histamine receptor H2 (*HRH2*), and *HTR7*, may reflect transcript-level changes in components linked to Ca²⁺-mobilising signalling. Importantly, irisin is known to stimulate LH and FSH secretion directly from pituitary gonadotrophs in bovine primary cultures^31^ and boosts LHβ/FSHβ mRNA stability in tilapia pituitary cells^26^. Considering that GnRH‑induced LH/FSH exocytosis depends on rhythmic Ca^2+^ oscillations^77,78^, the modulation of voltage-gated calcium channel (VGCC) subunits and related receptors by irisin may point to a possible relationship between irisin signalling and gonadotrophin release. By affecting Ca^2+^ entry pathways and the expression of receptors linked to Ca^2+^-mobilising ligands, irisin may potentially influence the threshold for GnRH-induced Ca^2+^ spikes. The observed transcriptional changes suggest that irisin regulates not only calcium influx but also intracellular signalling processes within the pituitary. Such modulation could critically influence both gonadotrophins and PRL secretion, processes that are essential during early gestation in pigs, when precise hormonal regulation is required to support embryo implantation and maintain corpus luteum function.

In addition to protein-coding genes, our analysis revealed extensive irisin-induced regulation of lncRNAs and AS events, highlighting multiple layers of transcriptional and post-transcriptional control. lncRNAs are pivotal regulators of diverse cellular functions and biological processes. They influence gene expression at multiple levels, including epigenetic, transcriptional, and post-transcriptional regulation^79^. In our analyses, several lncRNAs were associated with genes involved in angiogenesis and vascular development, including *VEGFC* (*MSTRG.8840.1; MSTRG.8838.1*) and collagen and calcium binding EGF domains 1 (*CCBE1*) (*MSTRG.1935.1; MSTRG.1933.1; MSTRG.1933.2; MSTRG.1933.3*). *VEGFC* is a key regulator of angiogenesis, endothelial cell proliferation, and lymphangiogenesis^80^, while *CCBE1* encodes a highly conserved protein indispensable for lymphatic development^81^. In zebrafish embryos, *CCBE1* functions as an integral component of the VEGFC/VEGFR3 signalling pathway^81^. Moreover, *VEGFC* is expressed in the normal human pituitary, suggesting a role in regulating intra-pituitary vascular permeability^82^. Our findings may indicate that irisin is associated with changes in the expression of specific lncRNAs linked to these processes. However, these observations should be considered exploratory, as no strongly correlated DEL–DEG pairs were detected.

Functional enrichment analysis revealed that lncRNAs were most significantly enriched in the MAPK signalling pathway, with up to nine lncRNA-associated genes identified. Notably, irisin decreased the expression of lncRNAs linked to *MAPK1* and *MAPK10*. The MAPK signalling cascades are essential mediators of extracellular signals, such as growth factors and cytokines, into intracellular responses, controlling processes such as cell growth, differentiation, and apoptosis^83^. In the pituitary, MAPK signalling is central to hormone secretion and cell proliferation^84^. For example, GnRH activates MAPK pathways, including ERK, JNK, and p38, in gonadotrophs^85^, thereby promoting LH and FSH production, which are critical for reproductive function^86^. Supporting this, Xiao et al.^87^ identified *MAPK1* and *MAPK10* as potential regulators of reproductive physiology in sheep, proposing that they modulate PRL and LH secretion, ultimately influencing follicular development and oocyte maturation. In the present study, exploratory enrichment placed several DEL-associated genes within the MAPK pathway, which may point to a possible relationship between irisin-responsive lncRNAs and MAPK-related signalling in porcine pituitary cells. Given the absence of strongly correlated DEL–DEG pairs, these observations should be interpreted as exploratory and not indicative of direct regulatory relationships.

AS provides an additional regulatory layer, enabling a single gene to generate multiple mRNA isoforms with distinct biochemical properties and functional outcomes^88,89^. In this study, we identified several AS events in APc treated with irisin. Notably, one involved the *MAP2K5* gene, also known as *MEK5*, a component of the MEK5/ERK5 signalling pathway that regulates cell growth, differentiation, and proliferation^90^. AS of *MAP2K5* produces two isoforms: *MEK5α*, associated with particulate cellular fractions, and *MEK5β*, localised in the cytosol^91^. Given the high specificity of MEKs-ERK interactions, AS may enhance signalling precision by directing distinct isoforms to particular cellular compartments^91^. Thus, irisin’s modulation of *MAP2K5* splicing may be associated with altered regulation of the ERK5 pathway in pituitary cells. The identified *MAP2K5* splicing event points to a candidate target for future functional validation. We also observed that irisin significantly affected the splicing of the *SMAD1* gene, where an SE event (ΔlncLevel = − 0.389) suggested the generation of an isoform with altered functional domains and potentially modified translational activity. SMAD1 is a key mediator of bone morphogenetic proteins (BMPs) and transforming growth factor-beta (TGF-β), which regulate anterior pituitary hormone expression and secretion^92^. Upon activation, SMAD1 is phosphorylated by BMP receptors and translocates to the nucleus, where it drives the transcription of gonadotrophin subunits *LHβ* and *FSHβ*^93^. Notably, BMP-4 has been shown to modulate *FSHβ* and the GnRH receptor (*GnRHR*) expression in gonadotrophs, thereby downregulating hormone secretion^94^. Therefore, altered *SMAD1* splicing may potentially affect BMP-mediated signalling, which in turn could influence gonadotroph responsiveness to hypothalamic GnRH and the regulation of pulsatile FSH release. Collectively, these findings suggest that irisin treatment may be associated with pituitary function not only at the transcriptional level but also through post-transcriptional mechanisms such as AS. Previous studies on tilapia and bovine have demonstrated that irisin modulates gonadotrophin secretion both centrally and peripherally^26,30.^ Our work identifies AS candidates potentially relevant to the reproductive axis.

## Conclusions

This study provides novel insights into the multifaceted role of irisin in regulating reproductive functions at the level of the anterior pituitary in pigs during early pregnancy. High-throughput transcriptomic profiling revealed that irisin treatment was associated with altered expression of a wide spectrum of protein-coding genes, lncRNAs, and AS transcripts, many of which were linked to in endocrine signalling, hormone secretion, cellular remodelling, and angiogenesis. Enrichment analysis suggested a possible relationship between irisin and GH/PRL-related pathways, as well as regulators of gonadotroph activity, indicating that this adipomyokine may participate in shaping the pituitary molecular environment during gestation. Moreover, altered expression of genes linked to vascular development and calcium signalling may point to a broader association of irisin with pituitary cell function and plasticity. To our knowledge, this is the first comprehensive transcriptomic overview of irisin action in the pituitary gland, providing a basis for further studies on myokine–endocrine interactions potentially involved in pituitary adaptation to pregnancy. Importantly, these results should be interpreted within the context of transcriptomic analysis and therefore represent potential regulatory mechanisms rather than functionally validated effects. Nonetheless, this study establishes a valuable molecular framework for understanding adipomyokine–pituitary interactions and provides a foundation for future mechanistic and functional investigations.

## Supplementary Information

Below is the link to the electronic supplementary material.

Supplementary Table S1

Supplementary Figure 1

Supplementary Table S2

Supplementary Table S3

Supplementary Table S4

Supplementary Table S5

Supplementary Figure 2

Supplementary Table S6

Supplementary Table S7

## Data Availability

The sequencing data from the study have been submitted to the European Nucleotide Archive database (https://www.ebi.ac.uk/ena) under accession number: PRJEB98233.

## Abbreviations

18S rRNA: 18 S ribosomal RNA
A3SS: Alternative 3′ splice sites
A5SS: Alternative 5′ splice sites
ACTB: Beta-actin
ADAM32: ADAM metallopeptidase domain 32
ANG: Angiogenin
ANGPT1: Angiopoietin 1
ANGPTL2: Angiopoietin-like 2
APc: Anterior pituitary cells
AS: Alternative splicing
BP: Biological processes
BMP: Bone morphogenetic protein
CACNA1F: Calcium voltage-gated channel subunit alpha1 F
CACNA1S: Calcium voltage-gated channel subunit alpha1 S
CC: Cellular components
CCBE1: Collagen and calcium binding EGF domains 1
cDNA: Complementary DNA
CNCI: Coding-Non-Coding Index
CPC: Coding Potential Calculator
CYSLTR1: Cysteinyl Leukotriene Receptor 1
DASs: Differentially alternative splicing
DEGs: Differentially expressed genes
DELs: Differentially expressed long noncoding RNAs
DKK4: Dickkopf-related protein 4
ECRG4: Oesophageal cancer-related gene 4
EFNA5: Ephrin-a5
ENA: European Nucleotide Archive
ER: Endoplasmic reticulum
ERK 1/2: Extracellular signal-regulated kinase 1 and 2
ERMARD: Early response to malaria and related diseases
FC: Fold change
FDR: False discovery rate
FNDC5: Fibronectin type III domain-containing protein 5
FSH: Follicle-stimulating hormone
GH: Growth hormone
GH1: Growth hormone 1
GnRH: Gonadotrophin-releasing hormone
GnRHR: Gonadotrophin-releasing hormone receptor
GO: Gene ontology
GSEA: Gene set enrichment analysis
HCRTR1: Hypocretin receptor 1
HPG: Hypothalamic-pituitary-gonadal
HRC: Histidine-rich calcium-binding protein
HRH2: Histamine receptor H2
HTR7: 5-Hydroxytryptamine (serotonin) receptor 7
IGF1: Insulin-like growth factor 1
IGFBP3: Insulin-like growth factor binding protein 3
IL-5: Interleukin 5
IL-7: Interleukin 7
IRF9: Interferon regulatory factor 9
IRS4: Insulin receptor substrate 4
JAK–STAT: Janus kinase-signal transducer and activator of transcription
KEGG: Kyoto encyclopedia of genes and genomes
LCN2: Lipocalin-2
LH: Luteinising hormone
lncRNAs: Long non-coding RNAs
MAPK: Mitogen-activated protein kinase
MAP2K5 (MEK5): Activated protein kinase kinase 5
MF: Molecular functions
MXEs: Mutually exclusive exons
P4: Progesterone
PCR: Polymerase chain reaction
PDGFRA: Platelet-derived growth factor receptor alpha
PDGFRB: Platelet-derived growth factor receptor beta
PDE1C: Phosphodiesterase 1 C
PGR: Progesterone receptor
PI3K/Akt: Phosphoinositide 3-kinase/protein kinase B
PRL: Prolactin
PRLR1: Prolactin receptor isoform 1
PTAFR: Platelet-activating factor receptor
qPCR: Quantitative PCR
RIN: RNA integrity number
RIs: Retained introns
RNA-Seq: High-throughput transcriptome sequencing
SAT1: Spermidine/spermine N1-acetyltransferase 1
SEs: Skipped exons
SHARPIN: Shank-associated rh domain interacting protein
SHC4: SHC adaptor protein 4
SPOCK3: Sparc/osteonetin, cwcv, and kazal-like domains proteoglycan 3
SST: Somatostatin
STAT1: Signal transducer and activator of transcription 1
SUPT20H: spt20 homolog, saga complex component
SV2C: Synaptic vesicle glycoprotein 2c
TGF-β: Transforming growth factor-beta
VEGFA: Vascular endothelial growth factor A
VEGFC: Vascular endothelial growth factor C
VGCC: Voltage-gated calcium channels
VOPP1: Vesicular, overexpressed in cancer, prosurvival protein 1
ZNHIT2: Zinc Finger HIT-Type Containing 2
