# Supplementary material for: Irisin modulates the transcriptomic profile of porcine anterior pituitary cells isolated from gilts on days 15–16 of pregnancy
Source: Sci Rep. 2026 May 12;16:21774. doi: 10.1038/s41598-026-51519-6 (PMC13357752; doi:10.1038/s41598-026-51519-6)
Supplement: Supplementary file 1 — Supplementary Table S1 [file 41598_2026_51519_MOESM1_ESM.docx]

**Supplementary Table 1. Primers and reaction conditions of qPCR and RT-PCR used for the validation of the obtained results**

| Primer target | Primer sequence (5′→3′) | Accession  number | Primer,  nmol/L | Conditions | Reference |
| --- | --- | --- | --- | --- | --- |
| Reference genes | | | | | |
| *18S RNA* | F:5’: TCCAATGGATCCTCGCGGAA -3'  R: 5’: GGCTACCACATCCAAGGAAG -3' | AY265350.1 | 400 | Activation: 95°C - 10 min;  40 cycles of:  Denaturation: 95°C - 15 s,  Annealing: 60°C for - 1 min  Elongation: 72°C for - 1 min | [44] |
| *ACTB* | F: 5’: ACATCAAGGAGAAGCTCTGCTACG-3’  R: 5’:GAGGGGCGATGATCTTGATCTTCA-3’ | U07786 | 200 |  | [86] |
| Differentially expressed genes (DEGs) | | | | | |
| *HTR7* | F: 5’: ACCGCTGGTATGCCATCTACCAC  R: 5’: GACTTTTGTAGCACAAGCTCAG | NM_214085.1 | 200 | Activation: 95°C - 10 min;  40 cycles of:  Denaturation: 95°C - 15 s,  Annealing: 60°C for - 1 min  Elongation: 72°C for - 1 min | This study |
| *HCRTR1* | F: 5’: GACCCTTCATCTGTGGCACT  R: 5’: TAGAGGTCATCTGCCCAGCGTTCA | AF097995 | 200 |  | [87] |
| *DKK4* | F: 5’: ACAACACGCTCGGCTAAGAG  R: 5’: GCCCAAACCAGCTTCTGTTC | XM_021077993.1 | 200 |  | This study |
| *PDGRFA* | F: 5’: CCTACATCGGCGTCACCTAC  R: 5’: CACCAGGTCCGAGGAGTCTA | NP_001302685.1 | 200 | Activation: 95°C - 10 min;  40 cycles of:  Denaturation: 95°C - 15 s,  Annealing: 62°C for - 1 min  Elongation: 72°C for - 1 min | This study |
| *ANGPT1* | F: 5’: CACGCTGAACGGTTACACAG  R: 5’: GACAGTTCCCGTCGTGTTCT | NM_213959.1 | 200 |  | This study |
| *LCN2* | F: 5’: ACAACGTCATCTCCACCCTG  R: 5’: GACACCACACGCACGACATA | NM_001244410.1 | 200 |  | This study |
| *ECRG4* | F: 5’: CCCTCGTGCGCAGTCCT  R: 5’: AGGCTGCTTAGGAACTCCTTG | NM_001244729.1 | 200 | Activation: 95°C - 10 min,;  40 cycles of:  Denaturation: 95°C - 15 s,  Annealing: 64°C for - 1 min  Elongation: 72°C for - 1 min | This study |
| *IGF1* | F: 5’: TCTACTTGGCCCTGTGCTTG  R: 5’: GAACTGAAGAGCGTCCACCA | NM_214256.1 | 200 |  | This study |
| Differentially expressed long noncoding RNA (DELs) | | | | | |
| MSTRG.8840.1 | F: 5’: CTTGCTGGGCTTCTGCTCT  R: 5’: ATCAACACTGGACACCGACC | MSTRG.8840.1 | 200 | Activation: 95°C - 10 min;  40 cycles of:  Denaturation: 95°C - 15 s,  Annealing: 60°C for - 1 min  Elongation: 72°C for - 1 min | This study |
| MSTRG.7703.1 | F: 5’: CTCTCCTTTTCTTCCGTGGGC  R: 5’: AGGGTTCTCTGGCAGTAGGT | MSTRG.7703.1 | 200 |  | This study |
| MSTRG.6385.1 | F: 5’: ATCGATCTCTCGGCTTTGAGG  R: 5’: CAGCGCCACAAAACTCCATC | MSTRG.6385.1 | 200 |  | This study |
| MSTRG.15172.1 | F: 5’: CCAAGAAGACCAGGAAACACAC  R: 5’: GCGGTTTTCTCCTCCACTCA | MSTRG.15172.1 | 200 |  | This study |
| MSTRG.17310.1 | F: 5’: CTGGTGATGGCCGTGTATGA  R: 5’: AGTTTGCCAGCAGTAGGGAC | MSTRG.17310.1 | 200 | Activation: 95°C - 10 min;  40 cycles of:  Denaturation: 95°C - 15 s,  Annealing: 62°C for - 1 min  Elongation: 72°C for - 1 min | This study |
| MSTRG.14618.1 | F: 5’: AACCTTGTCTCTGGCTGCAA  R: 5’: GTAGCCCTCGATCAGAACGG | MSTRG.14618.1 | 200 |  | This study |
| MSTRG.20340.1 | F: 5’: CACAGCCTGCCAAACAGTTC  R: 5’: GGGGACGTCTTTGGATCCTC | MSTRG.20340.1 | 200 | Activation: 95°C - 10 min;  40 cycles of:  Denaturation: 95°C - 15 s,  Annealing: 64°C for - 1 min  Elongation: 72°C for - 1 min | This study |
| MSTRG.16845.1 | F: 5’: ATACCAGCTGCACAGAGAGC  R: 5’: GCGCCTTATGGTCTGAGCTT | MSTRG.16845.1 | 200 |  | This study |
| MSTRG.7947.1 | F: 5’: GTTGCAGAGCAACAATAATACTGG  R: 5’: AGGCCCTATTTCACAGCAGG | MSTRG.7947.1 | 200 |  | This study |
| Differentially expressed alternative splicing events (DASs) | | | | | |
| *MAP2K5* | F: 5’: AAGAATTGCAGTGGCGGTTG  R: 5’: TTGGCTGTTTTCGCATGCAC | ENSSSCG00000004955 | 200 | Activation: 95°C - 10 min;  40 cycles of:  Denaturation: 95°C - 15 s,  Annealing: 60°C for - 1 min  Elongation: 72°C for - 1 min | This study |
| *SPOCK3* | F: 5’: CTCCATGAGCCCTGTCTGTG  R: 5’: CCGTGCACTGCTCATTCTTG | ENSSSCG00000009722 | 200 |  | This study |
| *SUPT20H* | F: 5’: CATTGCCCGGTACAATGCAG  R: 5’: GGCTGGTGTGCCTCTTTTCA | ENSSSCG00000009359 | 400 |  | This study |
| *SV2C* | F: 5’: GTACCTCGGGATGATGGTGG  R: 5’: TTCAGGCATGAACGTGAGGG | ENSSSCG00000034062 | 200 | Activation: 95°C - 10 min;  40 cycles of:  Denaturation: 95°C - 15 s,  Annealing: 62°C for - 1 min  Elongation: 72°C for - 1 min | This study |
| *ADAM32* | F: 5’: ACCCCAGTGGACTCTAGCAT  R: 5’: TCAGGTCCACATTGCAAAGCA | ENSSSCG00000015807 | 200 |  | This study |
| *SHARPIN* | F: 5’: GCCACCATGGAAGGACAGAA  R: 5’: TTCAACCGTGACCTGTAGCC | ENSSSCG00000005914 | 400 |  | This study |
| *VOPP1* | F: 5’: GCACAGAGGCCAAAAAGCAT  R: 5’: TTCACCACCTGCTCATACGG | ENSSSCG00000032734 | 400 |  | This study |
| *ERMARD* | F: 5’: GTGTGGTTTACACAGAATCAGGG  R: 5’: GACGTTGCGAAGGTTGAGTC | ENSSSCG00000004010 | 200 | Activation: 95°C - 10 min;  40 cycles of:  Denaturation: 95°C - 15 s,  Annealing: 64°C for - 1 min  Elongation: 72°C for - 1 min | This study |
| *EFNA5* | F: 5’: CGCTGATGTTTCTCGTGCTC  R: 5’: CGGCTGACTCATGTACGGTG | ENSSSCG00000036520 | 200 |  | This study |

*18S RNA* – 18S ribosomal RNA; *ACTB* – beta actin; *HTR7* – 5-hydroxytryptamine receptor 7; *HCRTR1* – hypocretin receptor 1; *DKK4* – dickkopf WNT signaling pathway inhibitor 4; *PDGFRA* – platelet derived growth factor receptor alpha; *ANGPT1* – angiopoietin 1; *LCN2* – lipocalin 2; *ECRG4* – ECRG4 augurin precursor; *IGF1* – insulin like growth factor 1; *MAP2K5* – mitogen-activated protein kinase 5; *SPOCK3* – SPARC (osteonectin), cwcv and kazal like domains proteoglycan 3; *SUPT20H* – SPT20 homolog, SAGA complex component; *SV2C* – synaptic vesicle glycoprotein 2C; *ADAM32* – ADAM metallopeptidase domain 32; *SHARPIN* – SHANK associated RH domain interactor; *VOPP1* – VOPP1 WW domain binding protein; *ERMARD* – ER membrane associated RNA degradation; *EFNA5* – ephrin A5
