# Supplementary figures and images for: Irisin modulates the transcriptomic profile of porcine anterior pituitary cells isolated from gilts on days 15–16 of pregnancy
Source: Sci Rep. 2026 May 12;16:21774. doi: 10.1038/s41598-026-51519-6 (PMC13357752; doi:10.1038/s41598-026-51519-6)

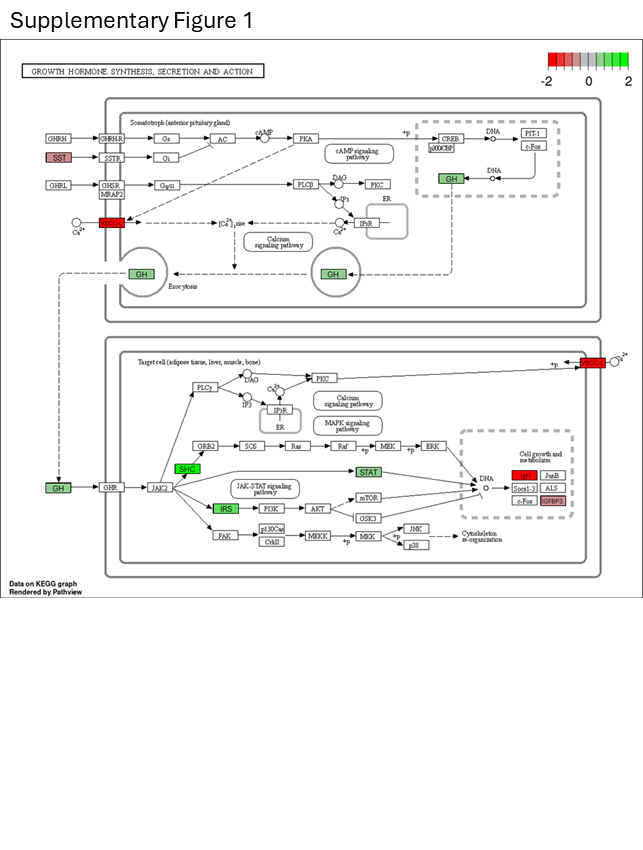

Supplement: Supplementary file 2 — Supplementary Figure 1 [file 41598_2026_51519_MOESM2_ESM.tif]

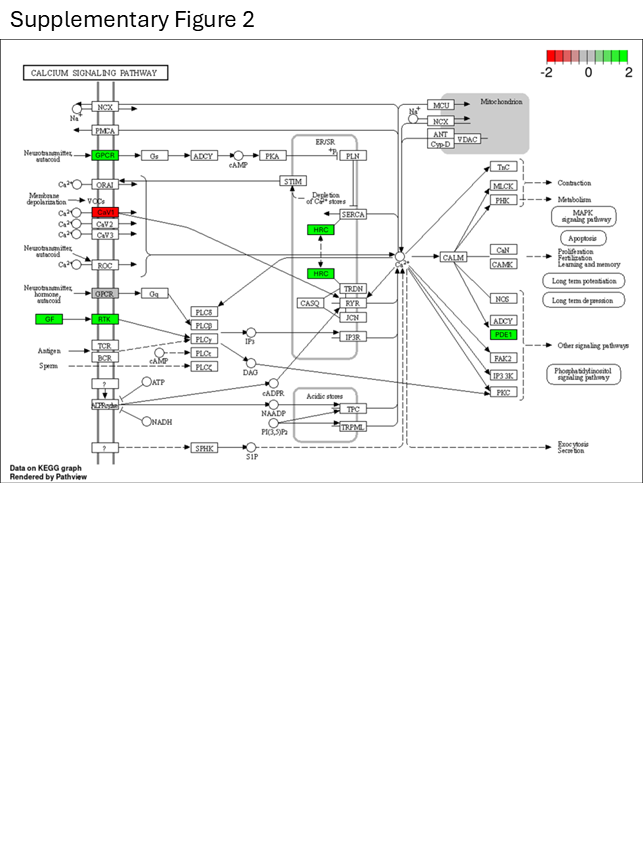

Supplement: Supplementary file 7 — Supplementary Figure 2 [file 41598_2026_51519_MOESM7_ESM.tif]
